# Coronary artery calcification progression and renal involvement in patients with systemic lupus erythematosus: a longitudinal cohort study

**DOI:** 10.1007/s00296-025-05785-8

**Published:** 2025-01-13

**Authors:** Lise Zinglersen, Amanda Hempel Zinglersen, Katrine Aagaard Myhr, Marie-Louise Hermansen, Klaus Fuglsang Kofoed, Andreas Fuchs, Louise P. Diederichsen, Søren Jacobsen

**Affiliations:** 1 Copenhagen Research Center for Autoimmune Connective Tissue Diseases (COPEACT), Copenhagen University Hospital, Rigshospitalet, Denmark; 2 Department of Cardiology, Copenhagen University Hospital, Rigshospitalet, Denmark; 3 Diagnostic Centre, Department of Rheumatology, Aarhus University Hospital, Aarhus, Denmark; 4 Department of Rheumatology, Odense University Hospital, Odense, Denmark

**Keywords:** SLE, Atherosclerosis, Coronary artery calcification progression, Impaired renal function, Myocardial infarction, Cardiovascular risk factors, Lupus nephritis

## Abstract

To investigate if progression of coronary artery calcification (CAC) in patients with systemic lupus erythematosus (SLE) is associated with renal and traditional cardiovascular risk factors as well as incidence of myocardial infarctions. CAC progression was evaluated by cardiac computed tomography (CT) at baseline and after 5 years. Multivariable Poisson regression was applied to investigate associations between CAC progression and baseline values for traditional cardiovascular risk factors, CAC, SLE disease duration, lupus nephritis, and renal function. Regarding renal function, three groups were defined based on eGFR. Further, we analysed association between CAC progression and myocardial infarction during follow-up. Of the 147 SLE patients, 99 had cardiac CT at baseline and 5-year follow-up, with a total of 502 patient-years. At baseline, their median age was 47 years, median SLE disease duration was 14 years, 88% were women, 58% had lupus nephritis, and the median eGFR was 99 mL/min/1.73m^2^. 38/99 (39%) had CAC progression. CAC progression was associated with smoking (ever) (relative risk [RR] 1.69, CI95% 1.19–2.40), SLE disease duration (RR per year 1.03, CI95% 1.01–1.04), and CAC presence (RR 2.52, CI95% 1.68–3.78) at baseline. During follow-up, myocardial infarction occurred in three (7.9%) CAC progressors and in two (3.3%) patients who did not have CAC at any time (RR 2.1, CI95% 0.0-5.5). In this study, progression of CAC was associated with smoking, SLE disease duration and the prior presence of CAC, but it was inconclusive as to associations with renal involvement and incidence of MI.

**Supplementary Information:**

The online version contains supplementary material available at 10.1007/s00296-025-05785-8.

## Introduction

Systemic lupus erythematosus (SLE) is a multiorgan, autoimmune disease. It is accompanied by an increased risk of cardiovascular disease (CVD) and cardiovascular mortality [1–4]. Even though the overall mortality of SLE is decreasing, this is not accompanied by lowered mortality due to CVD [4, 5]. Contributing factors to increased cardiovascular morbidity and mortality are the increased prevalence of traditional cardiovascular risk factors and accelerated arteriosclerosis seen in these patients [6, 7]. However, traditional CVD risk factors do not fully account for the increased CVD risk in SLE, and arteriosclerosis does not account for all cases of CVD in SLE patients [8–10]. This suggests that other factors, including disease-related factors, may contribute to the increased CVD risk in SLE patients [11].

Lupus nephritis (LN) is a common and severe manifestation of SLE which may evolve into end-stage renal disease in about one out of ten SLE patients [12–15]. LN is associated with an increased risk of myocardial infarction (MI) and cardiovascular mortality [16]. Previously, we have shown that LN combined with reduced renal function was strongly associated with coronary artery calcification (CAC) [17]. CAC and the progression of CAC is considered a well-established marker of coronary artery disease, including atherosclerosis and stenosis, and is predictive of future cardiac events in the general population [18–20].

CAC is increased [7] and also progresses over time in SLE patients [21, 22], but it has not yet been investigated if CAC or progression hereof correlates with future CVD events in SLE patients [23, 24].

Only two other studies have investigated predictors of CAC progression [21, 22]. However, these studies only followed patients for 2–4 years and did not investigate the association between CAC progression and clinical events, and changes in renal function over time.

The purpose of this 5-year follow-up study was thus to determine any associations between the progression of CAC and specific risk factors, including SLE-related renal involvement and traditional risk factors. In addition, we wanted to determine if CAC progression was associated with MI during the follow-up.

## Method

### Study cohort

Between June 2013 and May 2014, patients with SLE from Copenhagen Lupus and Vasculitis Clinic, Copenhagen University Hospital, Rigshospitalet, Denmark were included in the Prospective Lupus Study on Cardiovascular Disease (PLUSHEART) study [17]. The inclusion criteria were as previously described [17]. SLE condition was established by fulfilling 4 or more of the 1982 American College of Rheumatology criteria (ACR) including the 1997 revised criteria [25, 26]. The original cohort of patients was selected such that 50% of the cohort had LN. SLE disease duration was defined as the time from fulfilment of 4 or more ACR criteria to time of baseline inclusion.

Patients were reinvited after 5 years for a follow-up assessment, which took place between December 2018 and March 2019. All patients still alive were invited to participate, and all patients gave written informed consent.

The study programme was the same at baseline and follow-up, including questionaries, physical examinations, interviews, overnight fasting blood tests, and non-contrast cardiac CT [17]. Previous medication and diagnoses were retrieved from the patients’ medical records.

### Traditional cardiovascular risk factors assessment

The blood pressure was measured after a 5-minute resting period. Hypertension was defined as a systolic blood pressure > 140mmHg or diastolic blood pressure > 90mmHg, or any treatment with blood pressure lowering medication.

Hypercholesterolemia was defined as a total cholesterol ≥ 5 mmol/L or LDL ≥ 3 mmol/L or any treatment with statin.

Family-based risk assessment for cardiovascular disease (family CVD risk) was defined as a previous event of MI or stroke in a first-degree relative, for women before the age of 65 and for men before the age of 55 [27].

### Lupus specific assessment

LN diagnosis was biopsy-verified or based on the ACR criterion for renal involvement [25]. eGFR was calculated by the Cockcroft-Gault formula [28] with an estimated body surface area of 1.73m^2^.

To assess renal impairment, patients were divided into 3 groups based on the National Kidney Foundation Practice Guidelines for Chronic Kidney Disease [29]. The reference group (i) was patients with preserved renal function as defined by eGFR ≥ 90 mL/min/1.73m^2^. The other groups were (ii) patients with mild decreased renal function as defined by eGFR 60–89 mL/min/1.73m^2^, and (iii) patients with moderately or severely decreased renal function as defined by eGFR < 60 mL/min/1.73m^2^.

Disease activity was measured according to the Systemic Lupus Erythematosus Disease Activity Index 2000 (SLEDAI-2K) [30], and disease damage was measured according to the SLICC Damage Index (SDI) [31].

### Blood and urine analysis

A positive aPL was defined as an IgG or IgM anti-beta-2-glycoprotein1 antibody > 40 × 10^3^ IU/L and/or an IgG or IgM anti-cardiolipin antibody > 40 × 10^3^ IU/L and/or a positive lupus anticoagulant (LAC) test.

### CT imaging of the coronary arteries

The CT examinations were performed on a 320-multidetector CT scanner (Aquillon ONE, Canon Medical Systems, Otawara, Japan) according to institutional CT protocol, comprising non-contrast, prospectively ECG-gated cardiac CT. A cardio-selective beta-blocker was administered orally approximately one hour before scanning in patients with a heart rate > 60 bpm and no contraindications. Phases were reconstructed in 3.0 mm slice thickness with a 3.0 mm increment. Coronary artery calcium score image analyses were performed using commercial software (Vitrea 6.7, Vital Images, Minnesota, USA). The total amount of CAC was measured in volume (mm^3^).

CAC progression was defined as a difference of ≥ 2.5mm^3^ in the square volume of the total plaque in the coronary arteries between baseline and follow-up. This definition was used to account for interscan variability [32]. CAC presence is defined by any CAC volume score above zero.

### Statistical analysis

Categorical variables are presented as numbers (n) and percentages. Normally distributed variables are presented as mean, standard deviation (SD), and range; non-normally distributed variables as median, interquartile range [IQR], and range.

Non-parametric distributions were tested by the Mann-Whitney test for between-group testing and the Wilcoxon test for within-group testing. Normally distributed data were tested by independent t-tests for between-group testing and paired t-tests for within-group testing. Between-group testing for categorical values were tested using Pearson’s Chi-square or Fisher’s exact test, depending on whether the expected values were above or below 5, respectively. For within-group testing, categorical variables were tested by McNemar’s test, and only values that could change unexpectedly were analysed.

Univariate and age- and sex-adjusted Poisson logistic regression analyses were carried out to estimate relative risks (RR) of CAC progression by traditional cardiovascular risk factors (age, sex, smoking, hypertension, hypercholesterolemia, and family CVD risk at baseline), presence of baseline CAC, and certain SLE related factors (renal function, LN and SLE disease duration at baseline). The variables included was largely based on the baseline model. SLE disease duration was included as a surrogate endpoint of all disease and treatment related exposures. Baseline CAC was included to isolate only predictors of CAC progression. Diabetes mellitus was not included because of low prevalence (*n* = 4). A multivariable Poisson regression analysis fully adjusted for all the mentioned variables was performed to identify risk factors for CAC progression. The results are reported as RR with 95% confidence intervals (CI) and p-values.

In all aspects, two-sided P-values ≤ 0.05 were considered statistically significant.

IBM SPSS Statistics, version 28.0, was used for the statistical analysis.

## Results

5-year follow-up and cardiac CT-scan was carried out in 99 of the originally 147 SLE patients in the cohort. Causes for no-participation in follow-up were migration (three patients), failure to respond to invitation (12 patients), decline to participate (17 patients), declined CT-scan because of radiation exposure (11 patients), and one had an insufficient CT-scan. Furthermore, four patients died during the follow-up, one due to CVD. Thus, a total of 48 patients did not participate in follow-up CT-scans.

### Clinical characteristics and changes during follow-up

Patient characteristics at baseline and follow-up are presented in Table 1. Further baseline characteristics have been described previously [17].

**Table 1:** Characteristics of the systemic lupus erythematosus study cohort

Characteristics	Baseline (*n* = 99)	Follow-up (*n* = 99)	*p*§
Age, years mean [SD] (range)	47 [13] (21–71)	52 [13] (26–77)	-
Female sex, n (%)	87 (88)	87 (88)	-
Caucasian, n (%)	94 (95)	94 (95)	-
SLE disease duration years, median [IQR] (range)	14.3 [14] (0.1–41)	19.4 [14] (4.9–46)	-
SDI score median [IQR] (range)	1 [2] (0–8)	2 [3] (0–9)	**< 0.001**
SLEDAI-2K score median [IQR] (range)	4 [4] (0–28)	3 [2] (0–24)	**0.006**
aPL ever, n (%)*	58 (59)	60 (61)	-
LAC ever, n (%)	31 (31%)	35 (35%)	-
Glucocorticoids, ever, n (%)	95 (96)	98 (99)	-
Antimalarials, ever, n (%)	82 (83)	85 (86)	-
Other immunosuppressants, ever, n (%)	90 (91)	91 (92)	-
Lupus nephritis, n (%)	57 (58)	57 (58)	-
eGFR, mean [SD] (range)	99 [32] (30–185)	88 [29] (20–160)	**< 0.001**
eGFR > 90, n (%)	57 (58)	48 (48)	-
90 > eGFR > 60, n (%)	34 (34)	35 (35)	-
eGFR < 60, n (%)	8 (8)	16 (16)	-
Smoking, ever, n (%)	62 (63)	62 (63)	-
Smoking, pack years, median [IQR] (range)	9.0 [16] (0.3–72)	9.3 [17] (0.3–72)	**< 0.001**
Hypertension, n (%)	62 (63)	59 (60)	0.41
Hypercholesterolaemia, n (%)	62 (63)	75 (76)	**0.03**
Family CVD risk, n (%)	16 (16)	16 (16)	-
BMI, median [IQR] (range)	23.5 [6] (18–42)	24.2 [6] (18–45)	**0.003**
Diabetes mellitus, n (%)	4 (4)	6 (6)	0.16
Previous MI events, n (%)	3 (3)	8 (8)	-
CAC any, n (%)	37 (37)	51 (52)	**< 0.001**
CAC score, median [IQR] (range)	0.00 [47.0] (0.00-1036)	6.00 [104] (0-1340)	**< 0.001**

IQR: inter-quartile range; SD: standard deviation; SDI: SLICC Damage Index; SLEDAI-2K: Systemic Lupus Erythematosus Disease Activity Index 2000; aPL: antiphospholipid antibodies; LAC: Lupus anticoagulant; BMI: body mass index; CVD: cardiovascular disease; eGFR: estimated glomerular filtration rate (ml/min/1.73 m^2^); MI: myocardial infarction; CAC: coronary artery calcification

§Paired tests comparing baseline and follow-up. Statistical significance (*p* < 0.05) in bold; - not tested

*IgG or IgM anti-beta-2-glycoprotein-1-antibody > 40 × 103 IU/L and/or an IgG or IgM anti-cardiolipin-antibody > 40 × 103 IU/L and/or a positive lupus anticoagulants test

The mean follow-up time between baseline and follow-up CT-scans was 5.07 years (range 4.25–5.72). During this time, no further patients developed LN, but the mean eGFR decreased. Further characteristics are presented in Table 1.

The presence of LN was more common in the 99 patients studied compared with the 48 non-participating patients (58% vs. 35%, *p* = 0.006), but otherwise, the two groups did not differ (data not shown).

### Characteristics at follow-up of CAC progressors and non-progressors

At baseline, the median (IQR) CAC volume score was 0.00 (47), and 37 (37%) patients had presence of CAC. CAC increased at the follow-up to a median of 6.00 (104) with 51 (52%) patients having presence of CAC. Progression of CAC was observed in 38 (38%), of whom 11 (29%) did not have CAC at baseline.

The median CAC volume score at follow-up for progressors was 199, IQR [446], range (7-1340), and for non-progressors 0.0 IQR [0], range (0-1067).

Table 2 shows the characteristics of progressors and non-progressors at baseline and follow-up. At baseline, CAC progressors had longer SLE disease duration, higher SDI scores, lower SLEDAI-scores, and a lower eGFR, but aPL and LAC did not differ compared to non-progressors. The same SLE-related risk factors, except SLEDAI-scores, were also more pronounced for CAC progressors at follow-up. All traditional cardiovascular risk factors, except sex and family CVD risk, were more common amongst CAC progressors compared to non-progressors at baseline. At follow-up, only the traditional cardiovascular risk factors higher age, smoking, hypertension, and diabetes mellitus were more common amongst CAC progressors compared to non-progressors.

**Table 2:** Characteristics of 99 patients with systemic lupus erythematosus (SLE) stratified by progression of coronary artery calcification

	Baseline visit			Follow-up visit			Within progression groups	
Characteristics	Progressors (*n* = 38)	Non-progressors (*n* = 61)	p*	Progressors (*n* = 38)	Non-progressors (*n* = 61)	p**	*p*§	*p*§§
Age, mean (SD) ^a^	54 (10)	42 (13)	**< 0.001**	59 (10)	47 (13)	**< 0.001**	-	-
Female, n (%) ^c^	33 (87)	54 (89)	1.00	33 (87)	54 (89)	1.00	-	-
SLE disease duration, years median [IQR] ^b^	20.4 [15.5]	10.5 [13.1]	**< 0.001**	25.3 [15.5]	15.6 [12.7]	**0.004**	**-**	**-**
SDI, median [IQR] ^b^	2 [2]	1 [2]	**0.003**	3 [3]	1 [3]	**0.007**	**0.001**	**0.001**
SLEDAI-2K, median [IQR] ^b^	4 [4]	4 [6]	**0.035**	2 [4]	4 [2]	0.72	0.19	**0.011**
aPL ever, n (%)^ ^c^	26 (68)	32 (52)	0.12	26 (68)	34 (56)	0.21	-	-
Lupus nephritis, n (%) ^c^	21 (55)	36 (59)	0.71	21 (55)	36 (59)	0.71	-	-
eGFR, mean (SD) ^a^	89.7 (33.4)	105 (29.6)	**0.016**	76.5 (31.8)	94.7(24.8)	**0.002**	**0.001**	**0.001**
Smoking’, median [IQR] ^b^	8.38 [21.9]	0.75 [5.55]	**0.004**	8.55 [21.9]	0.75 [6.05]	**0.004**	**0.005**	**0.003**
Hypertension, n (%) ^c^	29 (76)	33 (54)	**0.026**	29 (76)	30 (49)	**0.007**	1.00	0.55
Hypercholesterolemia, n (%) ^c^	30 (79)	32 (52)	**0.008**	32 (84)	43 (70)	0.12	0.75	**0.019**
Family CVD risk, n (%) ^c^	9 (24)	7 (11)	0.11	9 (24)	7 (11)	0.11	-	-
BMI, median [IQR] ^b^	25.8 [7.05]	22.9 [4.52]	**0.003**	25.8 [5.82]	23.8 [5.64]	0.12	0.80	**0.001**
Diabetes mellitus, n (%) ^d^	4 (11)	0 (0)	**0.020**	5 (13)	1 (2)	**0.030**	1.00	1.00
Previous MI, n (%) ^d^	3 (8)	0 (0)	**0.026**	5 (13)	2 (3)	0.10	-	-

IQR: inter-quartile range; SD: standard deviation; SDI: SLICC Damage Index; SLEDAI-2K: Systemic Lupus Erythematosus Disease Activity Index 2000; eGFR: estimated glomerular filtration rate (ml/min/1.73m^2^); aPL: antiphospholipid antibodies; CVD: cardiovascular disease; BMI: body mass index: MI: myocardial infarction

^IgG or IgM anti-beta-2-glycoprotein-1-antibody > 40 × 103 IU/L and/or an IgG or IgM anti-cardiolipin-antibody > 40 × 103 IU/L and/or a positive lupus anticoagulants test, ‘ Pack-years

^a^ Normally distributed; ^b^ Nonparametric; ^c^ Categorical with all expected values above 5; ^d^ Categorical with expected values under 5

Statistically significant (*p* < 0.05) in bold, - not tested

Comparison of progressors and non-progressors at baseline (*) and follow up (**)

Comparison of baseline and follow-up for progressors (§) and non-progressors (§§)

In addition to Table 2, we tested the between-group difference of the change between baseline and follow-up, where only BMI (*p* = 0.02) was significantly higher for non-progressors. For both progressors and non-progressors, SDI and cumulative smoking increased during follow-up, whereas eGFR decreased. However, the between-group changes were not significant: SDI (*p* = 0.56), smoking (*p* = 0.17), and eGFR (*p* = 0.48) (data not shown). For non-progressors, SLEDAI decreased, whereas BMI and the prevalence of hypercholesterolemia increased during follow-up. The change in eGFR did not differ between patients with and without LN (*p* = 0.36) (data not shown).

### Regression model for progression of CAC

Table 3 shows the relationship between progression of CAC and both traditional cardiovascular risk factors and the SLE-specific risk factors (renal function, LN, and SLE disease duration) at baseline. The presence of CAC at baseline was associated with CAC progression with a RR of 2.52. In this fully adjusted model, age was not associated with CAC progression, but SLE disease duration was (RR = 1.03). Amongst the traditional cardiovascular risk factors for CVD, only a history of ever smoking was associated with CAC progression with a RR of 1.69, which can be transformed to an attributable proportion of 41% (95% CI: 16-58%). Neither LN nor renal function were independent predictors of progression in this fully adjusted model, although RR of CAC progression did tend to increase with increasing renal impairment (*p* = 0.06).

**Table 3:** Multivariable analysis of factors related to progression of coronary artery calcification (CAC) in 99 patients with systemic lupus erythematosus

Risk variables at baseline	CAC progression	
	RR (95% CI)	*p*
Age, years	1.00 (0.99–1.02)	0.27
Female sex (*n* = 87)	0.81 (0.49–1.33)	0.40
Smoking ever (*n* = 62)	**1.69 (1.19–2.40)**	**0.004**
Hypertension (*n* = 62)	0.86 (0.57–1.29)	0.45
Hypercholesterolaemia (*n* = 62)	1.38 (0.94–2.03)	0.10
Family CVD risk (*n* = 16)	1.11 (0.77–1.59)	0.58
CAC present at baseline (*n* = 37)	**2.52 (1.68–3.78)**	**< 0.001**
Renal function		
eGFR > 90 (*n* = 57)	1	
90 > eGFR > 60 (*n* = 34)	1.31 (0.90–1.89)	0.16
eGFR < 60 (*n* = 8)	1.69 (0.96–2.98)	0.07
LN (*n* = 57)	0.86 (0.61–1.20)	0.37
SLE disease duration, years	**1.03 (1.01–1.04)**	**0.001**

RR: relative risk; CVD: cardiovascular disease; eGFR: estimated glomerular filtration rate (ml/min/1.73 m^2^); LN: lupus nephritis; CI: confidence interval. Statistical significance (*p* < 0.05) in bold

**Table 4:** Cardiovascular history and characteristics of 5 patients with history of myocardial infarction (MI) among 99 patients with systemic lupus erythematosus before and after 5 years follow-up

Sex	Age at baseline	Events before baseline	Events during follow-up	CAC volume score at baseline	CAC volume score at follow-up	CAC progression	CKD class	LN class	Postive aCL and/or aB2G1	Postive LAC
Male	30 yrs	-	35 yrs, MI, LAD thrombosis (MRI)	0	0	No	2	IV	No	Yes
Male	40 yrs	33 yrs, MI, CAG not performed	45 yrs, MI, LAD thrombosis, no stenoses, PCI	2	20	Yes	2	-	No	Yes
Female	47 yrs	-	52 yrs, MI, no stenoses	0	0	No	1	-	No	Yes
Female	54 yrs	43 yrs, unstable angina, RCA+Cx PCI	56 yrs, MI, no stenoses	243	522	Yes	2	V	No	No
Female	66 yrs	-	68 yrs, stable angina;70 yrs, MI, LM CABG	266	1040	Yes	3	II	No	No

CAC: coronary artery calcium; CKD: chronic kidney disease; LN: Lupus nephritis; ACL: anti-cardiolipin autoantibodies; aB2G1: anti-Beta-2-glycoprotein-1 autoantibodies; LAC: lupus anti-coagulant; MI: myocardial infarction; CAG: coronary arteriography; LAD: left anterior descending artery; RCA: right coronary artery; Cx: circumflex artery; LM: Left main coronary artery; CABG: coronary artery bypass graft; PCI: Percutaneous coronary intervention; MRI: Magnetic resonance imaging; Yrs: years

Only one patient had eGFR < 30 mL/min/1.73m^2^ and no patients had eGFR < 15 mL/min/1.73m^2^ at baseline.

In the supplementary table (S1), the univariate and the age- and sex-adjusted analyses are presented, which show that the fully adjusted model is in coherence with the univariate and age- and sex-adjusted models. However, eGFR < 60 mL/min/1.73m^2^ is associated with CAC progression in the univariate and age- and sex-adjusted analyses.

### Cardiovascular event characteristics

A total of 7 patients in this cohort had a history of MI at follow-up. The incidence of MI during follow-up was 5 cases during 502 patient-years of follow-up, i.e., a mean value of 1 per 100 patient-years, (95% CI: 0.4–2.3); two of whom were non-progressors (3.3%) and three were CAC-progressors (7.9%) (RR = 2.1, 95% CI: 0.8–5.5, adjusted for age, sex, and smoking). These two CAC non-progressors did not have CAC at either baseline or follow-up but were characterized by having positive LAC and age below 50 years (Table 4). Invasive coronary angiography at the time of MI showed thromboses and no coronary artery stenoses in both cases, and neither were treated with invasive coronary treatment.

Among progressors with MI during follow-up, one patient had a low amount of CAC at follow-up (CAC volume score 20) and an invasive coronary angiogram with no stenoses, but signs of thrombosis. He was under the age of 50 and had positive LAC, and this patient was treated with percutaneous coronary intervention.

The two patients with the most CAC progression and MI were both smokers, had hypertension, hypercholesterolemia, and renal involvement, and were above 50 years, but had no LAC or aPL. One of these patients was treated with coronary artery bypass graft. For further details see Table 4.

## Discussion

In this prospective study, more than one-third of the SLE patients had progression of CAC over a five-year period, which, however, was not related to LN or the moderately impaired renal functions observed in this study. Instead, we found that the progression of CAC was associated with longer SLE disease duration, history of smoking, and previous presence of CAC. However, we also demonstrated that CAC was not a prerequisite for developing MI in these patients. Our analyses included a fully adjusted model showing the independent contribution of the studied risk factors for CAC progression.

### Renal involvement

The presence of CAC has been associated with impaired renal function, but it was not reported if LN influenced this result [33]. In our cohort, CAC presence at baseline was associated with LN and impaired renal function [17], and in univariate analyses we found that eGFR below 60 mL/min/1.73m^2^ was associated with CAC progression. However, this was not seen in the fully adjusted model, which may be explained by other risk factors influencing CAC progression, statistical power, and follow-up time. We found no association between LN and CAC progression in any of our analyses of CAC progression.

Only one other study investigating CAC progression included renal function and found no association with renal function nor with SLE disease duration. However, the 2-year follow-up may have been too short to allow detection of CAC progression [22]. Different definitions of CAC progression may also be a source for varying results, as it has previously been shown [34].

Patients with severely impaired renal function and dialysis patients have an increased risk of arterial calcification, seen in both the arterial intima and media layer of the arteries. Calcification in these patients is poorly correlated with obstructive coronary artery disease [35, 36]. The complete mechanism of this it not understood, nor is the influence of renal disease on CAC in SLE patients and its related CVD risk.

### Smoking

It is well-known that smoking affects cardiovascular health in both the general population and in SLE patients [22, 37, 38]. In this cohort, smoking was associated with CAC presence at baseline [17] as well as CAC progression during a 5-year follow-up, indicating that smoking is associated with the initial onset of CAC and contributes to the risk of CAC progression, approximately 40% in this study. CAC progressors as well as non-progressors had an increase in cumulative smoking during follow-up, however, this did not differ between the two groups. This may indicate that even if SLE patients quit smoking, they would still be at risk of CAC development within the first five years of smoking cessation. Whether smoking cessation influences CAC progression beyond a 5-year follow-up is unknown.

### SLE disease duration

We found that age was not associated with progression of CAC when adjusted for SLE disease duration. In the baseline study [17], age was associated with CAC presence, but this was not adjusted for SLE disease duration. Other studies found that SLE disease duration may independently correlate with CAC [39]. We consider SLE disease duration as a surrogate endpoint for potential exposure to disease-related risk factors. Hence, the fully adjusted model included disease duration to quantify all the disease-related factors that might influence CAC progression. Further investigation is needed to uncover what these risk factors are.

### Cardiovascular disease

Incidence of MI during follow-up was around 1% per year, which is in line with previous findings in Swedish SLE patients [6]. We were not able to demonstrate that CAC progression was associated with the incidence of MI, possibly due to the low number of events. Our study shows that some MI events in SLE patients are related to atherosclerosis and others are related to thrombosis that may be associated with aPL or other SLE-specific factors. This may imply that CAC may have limitations in the prediction of MI in patients with SLE.

The two SLE patients who experienced MI without the presence of CAC had in common a positive LAC. In a study of 16 SLE patients with acute MI, 5 patients had normal coronary angiograms, and 11 patients had coronary thrombosis. This patient group had a higher prevalence of aPL, more disease activity, and a shorter SLE disease duration at the time of the event compared to patients who suffered acute MI because of coronary aneurysm or atherosclerosis [10]. This might relate to disturbed microcirculation and thrombosis [40, 41]. Further investigation is needed to find out what causes these cases of CVD, and how CVD relates to CAC and aPL in SLE patients, especially regarding the influence of LAC.

### Implications and clinical relevance

CAC measures the calcification of plaques, which only makes up about one fifth of the total plaque [42, 43]. Furthermore, the presence of CAC does not specify if the plaque is protruding into the artery lumen and thus causing stenosis [44]. In the general population, CAC has been established to predict coronary heart disease [19], but this has not been investigated in SLE patients.

A metanalysis of CVD in SLE patients suggests SLE to be accounted as a risk factor for CVD equal to diabetes mellitus and thus SLE patients may require more extensive coronary health management [11, 45]. Furthermore, CAC is seen at earlier onset in SLE patients compared to the general population, emphasizing the need for early check-ups and prevention [7]. Even though CAC scores did not predict all cases of MI in our SLE patients, CAC score still seems to be an important assessment in SLE with 38% of patients having CAC progression over five years.

Based on our analysis, CAC progression was predicted by the CAC presence at baseline, and therefore such SLE patients might benefit from rescans. No other studies have investigated this in an SLE population, and further investigation is needed to establish this. The fact that presence of CAC is associated with progression of CAC might indicate which SLE patients are at higher risk of accelerated progression of CAC and can thus be used in a risk assessment. Based on the findings in this study, we suggest that physicians should particularly focus on patients with risk factors such as smoking, previous CAC findings, and long disease duration.

### Strengths and limitations

A strength of our study is that this is the longest study of CAC progression to date in SLE with CT-scans at baseline and follow-up. Further, the progression of CAC was adjusted for the presence of CAC at baseline in our fully adjusted regression model to show results for factors contributing to only progression.

The limited cohort size may have implications when reviewing the MI events in the cohort, with a low count of new cases occurring during follow-up. Our results in this regard should therefore only be considered indicative for MI assessment.

We included SLE disease duration as a proxy for disease and treatment related exposures. We have no means of measuring the exposures previous to study start, but it seems that disease duration is a risk factor in itself. However, without controls from the general population, we cannot estimate the specific attribution of disease related exposure.

To increase statistical power in the original cohort, patients were selected to include an equal proportion of patients with and without LN to study the primary interest, association between CAC and LN. No new cases of LN occurred during follow-up, but more patients in the follow-up group had LN compared to the non-participants, making the proportion of patients with LN at follow-up 58%. This prevalence is almost 4 times higher than seen in a population-based cohort of Danish SLE patients [12]. This skewing may compromise the generalisability of our findings. A strength in this aspect is the fact that baseline characteristics were available for all the originally included patients [17] and statistical analyses not shown, indicate that the 48 non-participating patients, did not differ in any other characteristics.

## Conclusion

In this longitudinal cohort study, progression of CAC was associated with smoking, SLE disease duration, and the prior presence of CAC, but the study was inconclusive as to associations between renal involvement and incidence of MI. However, the findings support current views of the importance of early prevention of CAC and reduction of traditional and disease-related cardiovascular risk factors in patients with SLE.

## Electronic supplementary material

Below is the link to the electronic supplementary material.

Supplementary Material 1

## Data Availability

The datasets collected for this manuscript are available from the corresponding author upon reasonable request.

## Declarations

### Note on related congress abstracts and publication

This work has previously been presented as a poster presentation at the 39th Scandinavian Congress of Rheumatology, in Copenhagen, Denmark, 2023. It was presented as a poster titled: “*Coronary artery calcium progression and renal involvement in patients with systemic lupus erythematosus: a longitudinal cohort study”* by Lise Zinglersen. The Abstract has been published in the Scandinavian Journal of Rheumatology: 10.1080/03009742.2023.2233371 abstract number: PP30.
