# Supplementary material for: Coronary artery calcification progression and renal involvement in patients with systemic lupus erythematosus: a longitudinal cohort study
Source: Rheumatol Int. 2025 Jan 13;45(1):26. doi: 10.1007/s00296-025-05785-8 (PMC11729070; doi:10.1007/s00296-025-05785-8)
Supplement: Supplementary file 1 — Supplementary Material 1 [file 296_2025_5785_MOESM1_ESM.docx]

**Coronary artery calcification progression and renal involvement in patients with systemic lupus erythematosus: a longitudinal cohort study**

*Lise Zinglersen^[[1]](#footnote-1)^, Amanda Hempel Zinglersen, Katrine Aagaard Myhr^[[2]](#footnote-2)^, Marie-Louise Hermansen^[[3]](#footnote-3)^, Klaus Fuglsang Kofoed ^2^, Andreas Fuchs^2^, Louise P. Diederichsen^1,4^, Søren Jacobsen.*

**Supplementary table 1 (S1): Univariable and sex- and age adjusted analyses of risk factors of progression of coronary artery calcification in 99 patients with systemic lupus erythematosus**

| **Risk variables at baseline** | **Univariable** | | **Sex and age adjusted** | |
| --- | --- | --- | --- | --- |
|  | **RR (95% CI)** | **p** | **RR (95% CI)** | **p** |
| Age, years | **1.04 (1.03-1.06)** | **<0.001** | - | - |
| Female sex (n=87) | 0.90 (0.60-1.37) | 0.63 | - | - |
| Smoking, ever (n=62) | **1.69 (1.22-2.33)** | **0.001** | **1.66 (1.20-2.29)** | **0.002** |
| Hypertension (n=62) | **1.93 (1.38-2.69)** | **<0.001** | **1.61 (1.15-2.25)** | **0.006** |
| Hypercholesterolaemia (n=62) | **2.17 (1.54-3.05)** | **<0.001** | **1.58 (1.10-2.25)** | **0.01** |
| Family CVD risk (n=16) | **1.62 (1.16-2.25)** | **0.004** | **1.40 (1.00-1.95)** | **<0.05** |
| CAC present at baseline (n=37) | **4.24 (3.10-5.81)** | **<0.001** | **3.23 (2.26-4.60)** | **<0.001** |
|  |  |  |  |  |
| Renal function |  |  |  |  |
| eGFR>90 (n= 57) | 1 |  | 1 |  |
| 90>eGFR>60 (n= 34) | **1.72 (1.26-2.33)** | **<0.001** | 1.24 (0.88-1.75) | 0.22 |
| eGFR<60 (n= 8) | **2.70 (1.78-4.10)** | **<0.001** | **2.00 (1.25-3.19)** | **0.004** |
| LN (n=57) | 0.91 (0.69-1.21) | 0.53 | 1.29 (0.95-1.75) | 0.10 |
| SLE disease duration, years | **1.05 (1.04-1.06)** | **<0.001** | **1.04 (1.02-1.05)** | **<0.001** |

RR: relative risk; CVD: cardiovascular disease; eGFR: estimated glomerular filtration rate (ml/min/1.73 m^2^); LN: lupus nephritis; CI: confidence interval. Statistical significance (p<0.05) in bold.

1. Copenhagen Research Center for Autoimmune Connective Tissue Diseases (COPEACT), Copenhagen University Hospital, Rigshospitalet, Denmark [↑](#footnote-ref-1)
2. Department of Cardiology, Copenhagen University Hospital, Rigshospitalet, Denmark [↑](#footnote-ref-2)
3. Diagnostic Centre, Department of Rheumatology, Aarhus University Hospital, Denmark

   ^4^ Department of Rheumatology, Odense University Hospital, Denmark [↑](#footnote-ref-3)
